# Short-, mid-, and long-term complications after multisystem inflammatory syndrome in children over a 24-month follow-up period in a hospital in Lima-Peru, 2020–2022

**DOI:** 10.3389/fped.2023.1232522

**Published:** 2023-11-24

**Authors:** Giancarlo Alvarado-Gamarra, Matilde Estupiñan-Vigil, Raquel Garcés-Ghilardi, Jesús Domínguez-Rojas, Olguita del Águila, Katherine Alcalá-Marcos, Rafael Márquez Llanos, Lucie Ecker, Carlos R. Celis, Carlos Alva-Diaz, Claudio F. Lanata

**Affiliations:** ^1^ Department of Pediatrics, Hospital Nacional Edgardo Rebagliati Martins, Lima, Peru; ^2^ Instituto de Investigación Nutricional, Lima, Peru; ^3^ Red de Eficacia Clínica y Sanitaria, REDECS, Lima, Peru; ^4^ Instituto Nacional Cardiovascular “Carlos Alberto Peschiera Carrillo”—INCOR, Lima, Peru; ^5^ Grupo de Investigación Neurociencia, Efectividad Clínica y Salud Pública, Universidad Científica del Sur, Lima, Perú; ^6^ Servicio de Neurología, Departamento de Medicina y Oficina de Apoyo a la Docencia e Investigación (OADI), Hospital Daniel Alcides Carrión, Callao, Peru; ^7^ Department of Pediatrics, School of Medicine, Vanderbilt University, Nashville, TN, United States; ^8^ Department of Epidemiology, London School of Hygiene and Tropical Medicine, London, United Kingdom

**Keywords:** COVID-19, death, hospitalization, long-term effect, pediatric multisystem inflammatory disease, Peru, multisystem inflammatory syndrome in children

## Abstract

**Objective:**

To determine the short-, mid-, and long-term complications after multisystem inflammatory syndrome in children (MIS-C) over a 24-month follow-up period in a hospital in Lima, Peru, 2020–2022, and to explore differences according to the immunomodulatory treatment received and type of SARS-CoV-2 virus circulating.

**Methods:**

Ambispective 24-month follow-up study in children <14 years of age diagnosed with MIS-C at the Hospital Nacional Edgardo Rebagliati Martins (HNERM).

**Results:**

A total of 62 children were admitted with MIS-C. The most common short-term complications and serious events were intensive care unit (ICU) admission, invasive mechanical ventilation (IMV) due to respiratory failure, and shock; predominantly during the second pandemic wave (lambda predominance) and in children that received intravenous immunoglobulin (IVIG) plus a corticosteroid. Two patients died during the first wave due to MIS-C. During prospective follow-up (median of 24 months; IQR: 16.7–24), only 46.7% of patients were followed for >18–24 months. Of the total, seven (11.3%) patients were identified with some sequelae on discharge. Among the 43 remaining children, sequelae persisted in five (11.6%) cases (neurological, hematological, and skin problems). Six patients (13.9%) presented with new onset disease (hematologic, respiratory, neurological, and psychiatric disorders). One patient died due to acute leukemia during the follow-up period. None of them were admitted to the ICU or presented with MIS-C reactivation. Two patients presented persistence of coronary aneurysm until 8- and 24-month post-discharge.

**Conclusion:**

In our hospital, children with MIS-C frequently developed short-term complications and serious events during the acute phase, with less frequent complications in the mid- and long-term. More studies are required to confirm these findings.

## 1. Introduction

Severe SARS-CoV-2 infections can occur in pediatrics, mainly due to multisystem inflammatory syndrome in children (MIS-C), which is a post-infectious clinical entity (1, 2). Short- and long-term complications of MIS-C have been reported in the United States and Europe, mostly involving persistent cardiac complications, with a small number of cases, and short follow-up periods (6–12 months post-discharge) (3–11). However, like other post-viral syndromes, MIS-C could be related to long-term organ damage and dysfunction in all body systems (12). On the other hand, studies reporting long-term complications are limited in South America and other low- and middle-income countries (LMICs) (3, 4, 13, 14).

It is necessary to identify and ensure transparency of complications post MIS-C since they can cause symptoms and signs that remain unresolved, limiting children's activities and reintegration into their daily lives (15). In addition, the frequency and severity of these complications could differ according to the variant of the circulating SARS-CoV-2 virus and the type of immunomodulatory treatment given in the acute phase (16–20). In this sense, we must be attentive to findings similar to other post-viral syndromes during follow-up, and be alert to sequelae, new diseases, rehospitalizations, and persistent cardiac involvement.

The objective of this study was to determine the short-, mid-, and long-term complications after MIS-C over a 24-month follow-up period in a hospital in Lima, Peru, 2020–2022, and to explore differences according to the immunomodulatory treatment received and type of SARS-CoV-2 virus circulating.

## 2. Materials and methods

### 2.1. Design and population

This was an ambispective study. MIS-C cases, according to the Center for Disease Control criteria (21), occurring in children <14 years of age were identified by a retrospective analysis of children hospitalized at the Hospital Nacional Edgardo Rebagliati Martins (HNERM), in Lima, Peru, from April 2020 to April 2022. The prospective follow-up study was up to 24 months. All patients diagnosed with MIS-C during the study period were included. Each included case was reviewed by a pediatrician (GAG, MEV, and RGG), a pediatric infectious disease specialist (OdA), a pediatric cardiologist (KAM and RML), and a pediatric critical care specialist (JDR).

During this period, Peru was affected by three COVID-19 waves: the first was from March 2020 to December 2020 and was associated with the wild-type virus; the second wave was from January 2021 to June 2021, with the Lambda variant; and the third wave took place between January and April 2022, with the Omicron variant (22, 23).

### 2.2. Procedure

A retrospective medical record review was performed to identify and extract data on medical complications and severity due to MIS-C until discharge or death during hospitalization (short-term complications). The follow-up of discharged cases was up to 24 months (prospective), searched by new consultations or hospitalizations in medical records and/or by contact by telephone or in-person at 6, 12, 18, and 24 months after discharge (mid-term complications up to 12 months, and long-term follow-up >12–24 months). Epidemiological, clinical, and treatment variables were collected for both the initial hospitalization as well as follow-up events.

### 2.3. Study variables

Shock and respiratory failure with invasive mechanical ventilation (IMV) were considered as short-term complications. Shock was considered present when vasopressors were used. We reported admission to an intensive care unit (ICU) as severe due to MIS-C. We defined coronary aneurysm as a *z*-score ≥2.5 (24), and macrophage activation syndrome (MAS) according to the criteria proposed by Wang et al. (25).

Sequelae was defined as a medical condition that appeared in hospitalization for MIS-C and persisted during the follow-up. A new disease was any illness diagnosed by a medical doctor during the follow-up study that was not present before MIS-C.

Furthermore, we registered upper respiratory symptoms (cough, rhinorrhea, and sore throat), signs of respiratory distress (tachypnea, intercostal, subcostal, or suprasternal retractions, nasal flaring, grunting, and use of accessory muscles), as well as gastrointestinal (diarrhea, vomiting, nausea, and abdominal pain), mucocutaneous (rash, erythema palms and soles, conjunctivitis, and mucositis), neurologic (seizure and persistent disorder of consciousness), and osteoarticular findings (arthralgia). Patients with MIS-C were categorized into four clinical phenotypes: (1) Phenotype with Kawasaki Disease (KD) (complete or incomplete) without shock, (2) Shock phenotype (need for inotrope/vasopressor or fluid resuscitation >20 ml/kg) without KD, (3) Shock with KD phenotype, and (4) Phenotype with fever and inflammation (MIS-C not meeting shock or KD phenotype criteria, and clinically stable) (26, 27).

### 2.4. Statistical analysis

Data was collected in Microsoft Excel^R^ and analyzed using STATA v.16 (Stata Corp LP, College Station, Texas, United States). Numerical variables were reported using median and interquartile range (IQR), based on the non-normal distribution of the data. Categorical variables were expressed using absolute and relative frequencies. The Chi-squared or Fisher exact tests were used to compare categorical variables, considering the observed and expected frequencies. The Kruskal–Wallis test was used to compare more than two quantitative variables considering the methodological assumptions. We considered *p* < 0.05 as statistically significant.

### 2.5. Ethical aspects

The study protocol was approved by the Ethics Committee of the HNERM (Code number: 8296-2021-256). To evaluate events during hospitalization, informed consent was not requested because the information was collected directly from the medical records. To participate in the prospective follow-up study, written informed consent was obtained from both parents and assent in children >8 years. Confidentiality of the participants was always maintained during the study.

## 3. Results

**Figure 1:**
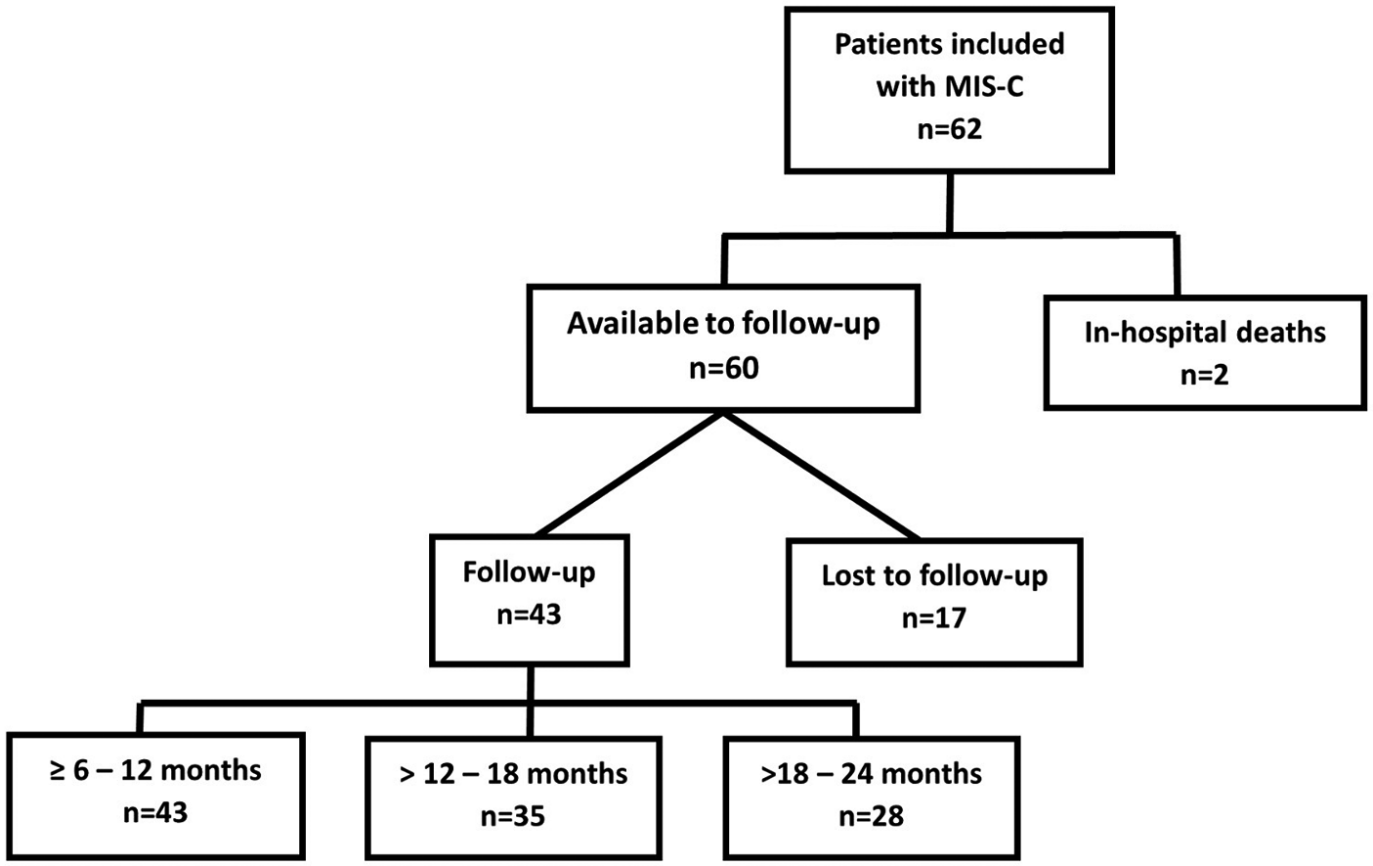
Flowchart of the participants with multisystem inflammatory syndrome in children (MIS-C) included in the study and duration of follow-up.

From the beginning of the SARS-CoV-2 pandemic until the end of the third wave, 62 patients were admitted to our hospital with a diagnosis of MIS-C (Figure 1), mainly during the first wave (54.84%). Most of the patients were male, of school age, the majority without comorbidities, and with a median illness duration of 5 days. Prior to MIS-C, three patients had received the COVID-19 vaccine (two doses). No data on nutritional status were recorded. Upon arrival at the emergency room, 17 patients (27.42%) presented O_2_ saturation of ≤92%, and seven (11.29%) required intubation. The most frequent MIS-C phenotype was shock with KD (32.26%), and the majority had gastrointestinal and mucocutaneous compromise (Table 1).

**Table 1:** Characteristics of patients with multisystem inflammatory syndrome in children (MIS-C) in a hospital in Lima-Peru, 2020–2022.

Characteristics	*n* (%) (*n* = 62)
Male	38 (61.29)
Age (years)^a^	7.42 (3.81–9.71)
Illness duration (days)^a^	5 (3–6)
Comorbidities	12 (19.35)
Previous intradomiciliary SARS-CoV-2 contact	26 (41.94)
SARS-CoV-2 epidemic waves^b^	
First wave: wild-type SARS CoV-2 virus predominance	34 (54.84)
Second wave: Lambda predominance	19 (30.65)
Third wave: omicron predominance	9 (14.52)
Clinical phenotypes	
KD (without shock)	15 (24.19)
Shock without KD	14 (22.58)
Shock with KD	20 (32.26)
Fever and inflammation	13 (20.97)
Clinical manifestations^c^	
Fever	62 (100)
Upper respiratory symptoms	39 (62.9)
Signs of respiratory distress	32 (51.61)
Gastrointestinal findings	50 (80.65)
Mucocutaneous findings	49 (79.03)
Neurologic findings	12 (19)
Osteoarticular findings	4 (6.45)

KD, Kawasaki disease.

^a^ Median (interquartile range).

^b^ First wave: March–December 2020 due to wild-type virus. Second wave: January 2021–June 2022, presumptively caused by Lambda variant. Third wave: from the first week of January to the first days of April 2022, Omicron variant predominance.

^c^ A patient could have one or more symptoms or findings. Upper respiratory symptoms: cough, rhinorrhea, and sore throat. Signs of respiratory distress: tachypnea, intercostal, subcostal, or suprasternal retractions; nasal flaring; grunting; and use of accessory muscles. Gastrointestinal findings: diarrhea, vomiting, nausea, and abdominal pain. Mucocutaneous findings: rash, erythema palms and soles, conjunctivitis, and mucositis. Neurologic findings: seizure and a persistent disorder of consciousness. Osteoarticular findings: arthralgia.

Furthermore, 59 patients (95%) had received some type of immunomodulatory treatment: 77.42% received intravenous immunoglobulin (IVIG) (2 g/kg) plus corticosteroids (2–30 mg/kg/day), 17.74% received IVIG alone, and 4.84% neither. No patient received anakinra, tocilizumab, or infliximab. In addition, the vast majority received acetylsalicylic acid (ASA) (91.94%), vasopressors/inotropes (54%), and antibiotics at the beginning of the clinical course (88.71%) (Supplementary Material Table S1).

### 3.1. Severity due to MIS-C and short-term complications

An important percentage of cases were admitted to the ICU (53.23%), with a median stay of 5 days. In addition, children frequently required IMV (40.32%) due to respiratory failure (median stay of 4 days) and developed shock (54%) while hospitalized (median length of hospitalization of 9 days). A smaller group required high-flow nasal cannula oxygen therapy (11.29%), developed MAS (12.9%), and presented coronary aneurysms (4.84%), and two patients died due to MIS-C (3.23%). ICU admission, IMV requirement, and shock were more frequent during the second wave (predominance of Lambda) (*p* < 0.05). MAS was more frequent during the third wave (predominance of Omicron) (*p* < 0.05). Two deaths (due to MIS-C) occurred during the first wave, an autopsy was not performed because the parents did not authorize it. On the other hand, ICU admission, IMV, and shock were more frequent in those who received IVIG plus corticosteroids. Hospitalization was longer in children who did not receive IVIG or corticosteroids (*p* < 0.05) (Table 2).

**Table 2:** Short-term complications in children with multisystem inflammatory syndrome (MIS-C) in a hospital in Lima-Peru, 2020–2022 (*n* = 62), according to the type of circulating SARS-CoV-2 virus and immunomodulatory treatment received.

Outcomes	SARS-CoV-2 epidemic wave predominance					Immunomodulatory treatment			
	Total *n* (%) (*n* = 62)	Wild type^a^ *n* (%) (*n* = 34)	Lambda^b^ *n* (%) (*n* = 19)	Omicron^c^ *n* (%) (*n* = 9)	*p*-value	IVIG only *n* (%) (*n* = 11)	IVIG plus corticosteroids *n* (%) (*n* = 48)	No IVIG or corticosteroids *n* (%) (*n* = 3)	*p*-value
Hospitalization length^d^	9 (7–13)	9 (6–15)	9 (7–13)	9 (8–11)	0.9843^h^	6 (3–8)	9.5 (7–14)	11 (6–14)	0.0224^h^
ICU	33 (53.23)	15 (44.12)	16 (84.21)	2 (22.22)	0.002^g^	0 (0)	32 (66.67)	1 (33.33)	<0.001^g^
ICU stay time^d^	5 (4–7)	5 (5–8)	5 (3.5–6)	5.5 (1–10)	0.5569^h^	0 (0)	5 (4–7.5)	5 (5–5)	0.7908^h^
IMV	25 (40.32)	12 (35.29)	12 (63.16)	1 (11.11)	0.002^f^	0 (0)	24 (50.0)	1 (33.33)	0.003^g^
IMV duration^d^	4 (3–5)	4.5 (3–5)	3 (3–5)	7 (7–7)	0.2616^h^	0 (0)	4 (3–5)	5 (5–5)	0.5202^h^
High-flow nasal cannula	7 (11.29)	4 (11.76)	3 (15.79)	0 (0)	0.555^g^	0 (11.76)	7 (14.58)	0 (0)	0.534^g^
MAS	8 (12.9)	3 (8.82)	0 (0)	5 (55.56)	0.001^g^	0 (0)	8 (16.67)	0 (0)	0.441^g^
Coronary aneurysm^e^	3 (4.84)	2 (5.9)	1 (5.3)	0 (0)	0.625^g^	0 (0)	3 (5.3)	0 (0)	0.235^g^
Shock	34 (54)	15 (44.12)	17 (89.47)	2 (22.22)	<0.001^g^	1 (9.09)	32 (6.67)	1 (33.33)	0.001^g^
Death (due to MIS-C)	2 (3.23)	2 (5.88)	0 (0)	0 (0)	0.658^g^	0 (0)	1 (2.08)	1 (33.33)	0.124^g^

IVIG, intravenous immunoglobulin; ICU, intensive care unit; IMV, invasive mechanical ventilation; MAS, macrophage activation syndrome; MIS-C, multisystem inflammatory syndrome in children.

^a^ First wave: March 2020–December 2020 caused by wild-type virus.

^b^ Second wave: January 2021–June 2021, presumptively caused by Lambda variant.

^c^ Third wave: from the first week of January to the first days of April 2022.

^d^ Median (interquartile range).

^e^ *Z* score ≥2.5.

^f^ Chi-square test.

^g^ Fisher exact test.

^h^ Kruskal–Wallis H test.

### 3.2. Mid- to long-term complications after MIS-C

Among the 60 remaining children, 17 (28.3%) were lost to follow-up (they could not be contacted by telephone and did not return to the hospital), of which 13 cases were from the first wave, two from the second wave, and two from the third wave. Of the remaining patients, 43 (71.7%) were followed for 6–12 months, however, eight patients did not continue due to the end of the study. Then, 35 (58.3%) patients were followed >12–18 months, however, seven patients also did not continue due to the end of the study. Finally, 28 (46.7%) patients were followed for >18–24 months (Figure 1). The median length of follow-up was 24 months (IQR 16.7–24), with a minimum follow-up of 6 months and a maximum of 24 months.

### 3.3. Sequelae

Of the total (*n* = 62), seven (11.3%) patients were identified with some sequelae on discharge, and two did not participate in follow-up. In these two patients, we noticed vascular necrosis of feet and hands in one patient, and right hemiparesis with gluteal decubitus ulcer in another (Supplementary Material Table S2). During follow-up (*n* = 43), sequelae persisted in five (11.6%) cases, of which four patients were from the first wave, and all received IVIG plus a corticosteroid during the acute phase (Table 3). Neurological sequelae (ischemic cerebrovascular disease with right hemiparesis and peripheral neuropathy of the left foot) and hematological problems (persistent pancytopenia due to MAS during hospitalization) were the most frequent conditions. Most of the sequelae persisted until 24 months, except for dermatological problems (urticaria) and persistent hair loss, which improved at 12 months (Supplementary Material Table S2).

**Table 3:** Mid- to long-term complications after multisystem inflammatory syndrome in children (MIS-C) in a hospital in Lima-Peru, 2020–2022 (*n* = 43), according to the type of circulating SARS-CoV-2 virus and immunomodulatory treatment.

Outcomes^a^	Total	Epidemic wave			Immunomodulatory treatment		
	*n* (%) (*n* = 43)	Wild type^b^ *n* (%) (*n* = 19)	Lambda^c^ *n* (%) (*n* = 17)	Omicron^d^ *n* (%) (*n* = 7)	IVIG only *n* (%) (*n* = 6)	IVIG plus corticosteroids *n* (%) (*n* = 35)	No IVIG or corticosteroids *n* (%) (*n* = 2)
Sequelae	5 (11.6)	4 (21.1)	0 (0)	1 (14.3)	0 (0)	5 (14.3)	0 (0)
New diseases	6 (13.9)	5 (26.3)	1 (5.9)	0 (0)	2 (33.3)	4 (11.4)	0 (0)
Death	1 (2.3)	1 (5.3)	0 (0)	0 (0)	0 (0)	1 (2.9)	0 (0)
Hospitalizations	6 (13.9)	6 (31.6)	0 (0)	0 (0)	2 (33.3)	4 (11.4)	0 (0)
Persistent coronary aneurysm^e^	2/42 (4.8)	1/18 (5.6)	1 (5.9)	0 (0)	0 (0)	2/34 (5.9)	0 (0)

IVIG, Intravenous immunoglobulin; ICU, Intensive care unit.

^a^ Mid-term complications up to 12 months, and long-term follow-up >12–24 months. Follow-up: 8 patients from 6 to 12 months, 7 patients >12 to 18 months, and 28 patients >18 to 24 months. The median time of follow-up was 24 months (IQR 16.7–24).

^b^ First wave: March 2020–December 2020.

^c^ Second wave: January 2021 to June 2021, presumptively caused by Lambda variant.

^d^ Third wave: from the first week of January to the first days of April 2022.

^e^ *Z* score ≥2.5. “*n*” total was 44, one of the three patients with coronary involvement could not be contacted for follow-up.

### 3.4. New diseases

Of the 43 patients, six patients (13.9%) presented with new onset disease, which was more frequent during the first wave and in those who received only IVIG (Table 3). One patient was diagnosed with acute lymphocytic leukemia (ALL) (1½ months post-discharge, no blasts in peripheral blood or bone marrow during hospitalization, and improvement of clinical status post-MIS-C). Other events that appeared were respiratory and neurological/psychiatric problems. Four patients presented three problems within a 12-month period (mid-term follow-up): leukemia, persistent tensional headache, and acute obstructive bronchial syndrome. Additionally, two problems appeared >12–24 months (long-term follow-up): anxiety disorder plus chronic abdominal or chest pain and acute obstructive bronchial syndrome (Supplementary Material Table S2).

### 3.5. Death, hospitalizations, ICU, and reactivation of MIS-C

One patient (from the first wave), who received IVIG plus corticosteroids during hospitalization, died 18 months after discharge due to ALL. Furthermore, six patients (13.9%) were re-admitted to the hospital, all from the first wave, and this was more frequent in those who received IVIG alone (Table 3). One patient was re-admitted due to ALL, while among the remaining five, one readmission was due to the moderate COVID-19 omicron variant, another was for a moderate acute bronchial syndrome, one for peritonitis (with chronic kidney disease prior to diagnosis of MIS-C), and two due to pancytopenia. No patient was admitted to the ICU or presented reactivation of MIS-C (Supplementary Material Table S2).

### 3.6. Persistent coronary aneurysm

Regarding coronary aneurysms, one of the three patients with coronary involvement could not be contacted for follow-up. In the other two, we observed aneurysm persistence (both asymptomatic, no hospitalizations required, and received treatment of 3–5 mg/kg/day of ASA) (Table 3). In one patient the coronary aneurysm persisted up to 8 months after discharge (echocardiography without alterations at 12 months), and in the other patient up to 24 months. Both received IVIG plus a corticosteroid in the acute phase (Supplementary Material Table S2). The remaining patients showed no evidence of coronary involvement in echocardiography, no systolic-diastolic dysfunction, no valvular regurgitation, or pericardial effusion during follow-up (8 patients with follow-up of 6 and 12 months, 7 patients with follow-up of 12–18 months, and 27 patients with follow-up of 18–24 months).

## 4. Discussion

This study found that there were frequent short-term complications and serious events during hospitalization due to MIS-C (ICU, IMV, and shock), especially during the second wave of the SARS-CoV-2 pandemic (predominant Lambda variant) and in patients who received IVIG plus corticosteroids. Long-term complications were less frequent but had an important impact on patients, with mainly hematologic, neurologic, and psychiatric sequelae and diseases. Most of these complications developed in patients infected during the first wave, who received different schemes of IVIG and corticosteroids. However, further studies are needed to confirm these findings.

### 4.1. Short-term complications

Our findings agree with other studies that reported frequent critical outcomes during hospitalization (1, 2). Moreover, in our hospital, we observed a higher impact of critical outcomes with the Lambda variant compared with the Omicron and wild-type virus. The first Lambda cases reported in Peru had an enormous impact on the country's public health (22, 23). A study conducted in Poland found no differences in the clinical course between different COVID-19 variants but reported few cases, and COVID-19 variants were not evaluated individually (16).

In addition, we found fewer patients with MIS-C during the Omicron wave compared with the other waves. Other studies have reported a lower incidence of MIS-C with Omicron compared with Delta (17, 19). With time, more children will become infected and vaccinated, and the incidence of MIS-C and its clinical impact will likely decrease. On the other hand, most patients with critical outcomes received IVIG plus corticosteroids, probably due to clinical severity. Moreover, in those who did not receive IVIG or corticosteroids, hospitalization was longer. Currently, there is no consensus about the use of immunomodulatory treatment, highlighting the need for the development of randomized controlled clinical trials in this respect (18, 28, 29).

### 4.2. Mid- and long-term complications

In our study, most patients presented a favorable long-term evolution, similar to the results published in other countries (3, 5, 30–38); however, there were some serious events during the follow-up, namely, sequelae, new diseases, death, hospitalizations, and persistent aneurysms. Otherwise, the follow-up of this study is one of the longest reported to date (up to 24 months), with other authors reporting up to 16 and 18 months (6, 38), but most with follow-ups of 6–12 months (3, 30–37).

Regarding the complications, two patients had persistent pancytopenia post-MAS (secondary to MIS-C). Others developed neurological sequelae, probably due to severe involvement during the acute MIS-C episode and as part of the post-ICU syndrome. Other studies have described two patients with peripheral digital gangrene bilaterally on their feet with a follow-up of 8 months (3), neuromuscular weakness and respiratory involvement of 4–5 months (3, 4), and resolution of renal, hematologic, and otorhinolaryngology alterations up to 6 months (5).

Furthermore, we describe a patient with ALL at 1½ months post-discharge, although this could be just a coincidence unrelated to MIS-C. Anxiety problems and persistent tension headaches have also been reported. Other studies have reported the persistence of neuropsychiatric symptoms (up to 18 months) (6), and dysfunction of the emotional/cognitive sphere, functional state (physical activity and basic cognition), muscle strength, and aerobic state (up to 4–6 months) (4, 5, 7). Long-term follow-up of these patients is necessary since MIS-C is a new clinical entity, characterized by not well defined hyper inflammation, with potential systemic complications, and the criteria used for diagnosis are under constant review (there is no confirmatory testing, and other proinflammatory systemic states can mimic the diagnosis) (39).

Regarding the COVID-19 pandemic waves, in our hospital, most patients with mid- and long-term complications were from the first wave (predominance of Wuhan variant). It is important to mention that there were more cases diagnosed during the first wave in our hospital and more studies are needed to confirm this finding. In the United States, in a study with a maximum follow-up of 6 months, no differences were found when evaluating outcomes according to the COVID-19 variant (comparing Wuhan, Alpha, and Delta) (40).

No participant was admitted to the ICU or had a reactivation of MIS-C during follow-up. Patients without coronary involvement did not develop aneurysms or other cardiac disorders during follow-up. Two patients persisted with coronary aneurysm up to 8 and 24 months after discharge. Most studies have reported persistence until 6–12 months (3, 31–33, 35, 37), while some describe persistence up to 16 months (38). They also report improvement in systolic and diastolic dysfunction at 6–12 months (30, 32, 34–38). Cardiovascular follow-up of the patients is important, especially in those who develop coronary abnormalities in the acute phase, similar to the recommendations for patients with Kawasaki disease (24).

### 4.3. Limitations and strength

About the limitations of the study, it is important to mention that 17 patients (28.3%) did not participate in the follow-up (13 patients from the first wave), and the 24-month follow-up was accomplished in approximately 46.7% of cases. The results are exploratory, considering the low retention rate, low number of cases per group, inadequate statistical power to detect differences, and patient outcomes from a single hospital. Furthermore, the events could be related to multifactorial problems during hospitalization and not just MIS-C. Finally, we cannot identify SARS-CoV-2 variants through genomic sequencing to explore differences among them. We consider the predominant variant per wave (greater than 80%), but other variants may also exist during that period. On the other hand, the main strength of this study is the length of cohort follow-up, which is the longest in Peru and probably in South America. Data from these patients could contribute to the understanding of the mid- and long-term complications of MIS-C. Additionally, the retrospective data were reviewed twice before analysis and we were able to verify the findings during the prospective follow-up.

In conclusion, MIS-C cases in our hospital (Lima, Peru) frequently presented short-term complications and serious events (ICU, IMV, and shock) during the acute phase, with the onset being more common during the second wave (Lambda predominance) and in children who received IVIG plus corticosteroids. Mid- and long-term complications were less frequent and were mainly due to hematologic, neurologic, and psychiatric disorders. Furthermore, persisting coronary aneurysms were found up to 8 and 24 months after discharge. Nonetheless, these are preliminary results and further studies are needed in other LMICs to confirm the frequency of the complications and clarify the impact of COVID-19 variants and immunomodulatory treatment.

## Data Availability

The raw data supporting the conclusions of this article will be made available by the authors, without undue reservation.
