# Supplementary material for: Short-, mid-, and long-term complications after multisystem inflammatory syndrome in children over a 24-month follow-up period in a hospital in Lima-Peru, 2020–2022
Source: Front Pediatr. 2023 Nov 24;11:1232522. doi: 10.3389/fped.2023.1232522 (PMC10710304; doi:10.3389/fped.2023.1232522)
Supplement: Supplementary file 1 [file Table1.doc]

Supplementary Material

# Supplementary Tables

**Supplementary Table 1. Treatment of children with multisystem inflammatory syndrome in a hospital in Lima-Peru, 2020-2022.**

| **Treatment** | **n (%)**  **(n=62)** |
| --- | --- |
| IVIG only | 11 (17.74) |
| IVIG plus corticosteroids | 48 (77.42) |
| No IVIG or corticosteroids | 3 (4.84) |
| Two IVIG doses | 15 (24.19) |
| Anakinra, tocilizumab, or infliximab | 0(0) |
| Acetilsalicilic acid | 57(91.94) |
| Vasopressors/Inotropes | 34(54) |
| Antibiotics | 55(88.71) |
| - Ceftriaxone | 26 (47.3) |
| - Meropenem plus vancomycin | 10 (18.2) |
| Ivermectin | 7(11.29) |
| Hydroxichloroquine | 4(6.45) |
| Anticoagulation | 5(8.1) |
| - Therapeutic | 4(6.45) |
| - Prophylactic | 1 (1.61) |

**IVIG**: Intravenous immunoglobulin.

# Supplementary Table 2. Characteristics of children with mid- to long-term complications after

**multisystem inflammatory syndrome in a hospital in Lima-Peru, 2020-2022.**

| **Patient** | **Age (years)** | **Sex** | **Follow-up (months)** | **SARS-CoV-2 vaccine before MIS-C ^a^** | **Critical outcomes** | **Type of predominant SARS-CoV-2 virus** | **Immunomodulatory**  **treatment** | **Sequelae** | **New diseases** | **Death** | **Hospitalizations** | **Persistent coronary aneurysm** | **Admitted to the ICU** | **Reactivation of MIS-C** |
| --- | --- | --- | --- | --- | --- | --- | --- | --- | --- | --- | --- | --- | --- | --- |
| 1 | 10 | M | 24 | No | ICU: No  IMV: No  Vasopressors: No | Wild type | IVIG plus corticosteroids | Persistent pancytopenia(due to MAS during hospitaliozation) ^b^, with portal hypertension and splenomegaly | No | No | Yes (due to pancytopenia) | No | No | No |
| 2 | 9 | F | 19 | No | ICU: Yes  IMV: Yes  Vasopressors:Yes | Wild type | IVIG plus corticosteroids | Ischemic cerebrovascular disease and right hemiparesis | Leukemia ^c^ (Diagnosis: 1½ months post discharge) | Yes (due to leukemia) | Yes (due to leukemia) | No | No | No |
| 3 | 9 | F | 24 | No | ICU: Yes  IMV: Yes  Vasopressors:Yes | Wild type | IVIG plus corticosteroids | Dermatological problems (urticaria) and persistent hair loss improving at 12 months after discharge. | Persistent tensional headache (Diagnosis: four months post discharge) | No | No | No | No | No |
| 4 | 11 | M | 24 | No | ICU: No  IMV:No  Vasopressors:No | Wild type | IVIG plus corticosteroids | Persistent pancytopenia(due to MAS during hospitaliozation) ^b^ | No | No | Yes (due to pancytopenia) | No | No | No |
| 5 | 9 | F | 9 | Si | ICU: Yes  IMV: Yes  Vasopressors: Yes | Omicron | IVIG plus corticosteroids | Peripheral neuropathy of the left foot | No | No | No | No | No | No |
| 6 | 7 | F | 0 ^d^ | No | ICU: Yes  IMV: Yes  Vasopressors: Yes | Wild type | IVIG plus corticosteroids | Vascular necrosis of feet and hands | NR | NR | NR | NR | NR | NR |
| 7 | 3 | M | 0 ^d^ | No | ICU: Yes  IMV: Yes  Vasopressors: Yes | Wild type | IVIG plus corticosteroids | Right hemiparesis, with gluteal decubitus ulcer | NR | NR | NR | NR | NR | NR |
| 8 | 9 | M | 24 | No | ICU: No  IMV: No  Vasopressors:No | Wild type | IVIG only | No | Anxiety disorder plus chronic abdominal pain or chest pain  (19 months post discharge) | No | Yes (moderate COVID-19 omicron variant) | No | No | No |
| 9 | 3 | M | 24 | No | ICU: No  IMV: No  Vasopressors:No | Wild type | IVIG only | No | Acute obstructive bronchial syndrome (14 months post discharge) | No | Yes (due to moderate acute bronchial syndrome) | No | No | No |
| 10 | 4 | M | 24 | No | ICU: No  IMV: No  Vasopressors:Yes | Wild type | IVIG plus corticosteroids | No | Persistent tensional headache (four months post discharge) | No | No | No | No | No |
| 11 | 0.3 | F | 19 | No | ICU: Yes  IMV: Yes  Vasopressors:Yes | Lambda | IVIG plus corticosteroids | No | Acute obstructive bronchial syndrome (8 months post discharge) | No | No | Yes ^e^ | No | No |
| 12 | 3 | M | 24 | No | ICU: No  IMV: No  Vasopressors:No | Wild type | IVIG plus corticosteroids | No | No | No | Yes(due to peritonitis) ^f^ | Yes ^g^ | No | No |

a.At least two doses.

b.A bone marrow aspirate was performed in which no blasts were found.

c.No blasts in peripheric blood or bone marrow during hospitalization and improvement of clinical status post MIS-C.

d.Retrospective data of medical record.

e.Coronary aneurysm persisted up to 8 months after discharge.

f.With chronic kidney disease prior to diagnosis of MIS-C.

g.Coronary aneurysm persisted up to 24 months after discharge.

**MIS-C**: Multisystem inflammatory syndrome in children. **ICU**: Intensive care unit. **IMV**: Invasive mechanical ventilation. **IVIG:** intravenous immunoglobulin. **NR:** Not reported.
